# Physics-Guided Neural Surrogate Model with Particle Swarm-Based Multi-Objective Optimization for Quasi-Coaxial TSV Interconnect Design

**DOI:** 10.3390/mi16101134

**Published:** 2025-09-30

**Authors:** Zheng Liu, Guangbao Shan, Zeyu Chen, Yintang Yang

**Affiliations:** Faculty of Integrated Circuit, Xidian University, Xi’an 710071, China; lzbest@sina.com (Z.L.); 24251215547@stu.xidian.edu.cn (Z.C.); ytyang@xidian.edu.cn (Y.Y.)

**Keywords:** neural network, causality, passivity, quasi-coaxial through-silicon-via (TSV), particle swarm optimization

## Abstract

In reconfigurable radio frequency (RF) microsystems, the interconnect structure critically affects high-frequency signal integrity, and the accuracy of electromagnetic (EM) modeling directly determines the overall system performance. Conventional neural network-based surrogate models mainly focus on minimizing numerical errors, while neglecting essential physical constraints, such as causality and passivity, thereby limiting their applicability in both time and frequency domains. This paper proposes a physics-constrained Neuro-Transfer surrogate model with a broadband output architecture to directly predict S-parameters over the 1–50 GHz range. Causality and passivity are enforced through dedicated regularization terms during training. Furthermore, a particle swarm optimization (PSO)-based multi-objective intelligent optimization framework is developed, incorporating fixed-weight normalization and a linearly decreasing inertia weight strategy to simultaneously optimize the S11, S21, and S22 performance of a quasi-coaxial TSV composite structure. Target values are set to −25 dB, −0.54 dB, and −24 dB, respectively. The optimized structural parameters yield prediction-to-simulation deviations below 1 dB, with an average prediction error of 2.11% on the test set.

## 1. Introduction

With the advancement of semiconductor-manufacturing technologies, through-silicon-via (TSV)-based three-dimensional (3D) integration has successfully overcome the interconnection bottlenecks of conventional planar designs. This technology can effectively shorten interconnection lengths, significantly increase chip interconnection density and communication bandwidth, and reduce signal transmission loss [1]. However, the geometric discontinuity in transitioning from horizontal interconnects to vertical TSVs introduces impedance mismatches and aggravates crosstalk, severely impairing signal integrity. Therefore, accurately evaluating the electromagnetic (EM) characteristics of TSV composite structures (e.g., S-parameters) has become a key prerequisite for optimizing the interconnect structures of reconfigurable radio frequency (RF) microsystems [2,3,4,5].

Current mainstream EM simulation approaches have inherent limitations. Full-wave EM simulations, although highly accurate, typically require substantial computational resources and long processing times [6]; equivalent circuit models perform well in low-frequency scenarios but fail to accurately capture EM effects at high frequencies, thus limiting their applicability [7]. In recent years, neural network-based surrogate models have been widely applied for predicting the EM performance of microsystems [8]. For example, Li et al. [9] employed a back-propagation (BP) neural network to predict the performance of TSV arrays.

Existing studies have primarily focused on improving the prediction accuracy of raw data (e.g., S-parameters or Y-parameters), often overlooking the fact that the predicted results must satisfy inherent physical constraints [10,11]. For multi-port passive microwave devices or interconnect structures, causality and passivity are intrinsic properties. Neglecting these characteristics may lead to prediction failures in subsequent multi-domain optimization or joint time–frequency simulations [12].

Torun et al. [13] proposed an improved spectral-transposed convolutional neural network (S-TCNN) that ensures causality and passivity in predicted S-parameter sequences, but its complex architecture demands a large amount of training data. In contrast, the neural transfer function (Neuro-TF) model [14] features a simpler structure and lower data requirements, but fundamentally fails to preserve physical consistency. This contradiction highlights a key challenge in current research: how to effectively embed physical constraints into neural networks while reducing dependence on large-scale training datasets, thereby achieving physically consistent predictions.

To address the challenge of broadband, physically consistent S-parameter prediction for TSV composite interconnects, we propose a physics-constrained Neural Transfer Function (Neuro-TF) surrogate together with a PSO-driven co-design framework. Methodology: (i) we embed causality and passivity regularizers into a three-stage training scheme (causality is enforced by constraining poles to the left half-plane, while passivity is promoted via penalties on the largest singular value of the scattering matrix); (ii) we incorporate vector-fitting priors (pole–residue form) to stabilize broadband learning and enhance interpretability; (iii) we formulate a multi-objective PSO with fixed-weight normalization and a linearly decreasing inertia weight to jointly optimize key geometry variables—TSV radius, insulation thickness, and signal–ground TSV pitch—under the co-objectives $S_{11}$, $S_{21}$, and $S_{22}$; and (iv) we establish an end-to-end pipeline spanning sample design (LHS), 1–50 GHz surrogate training, post hoc physical-consistency checks, and multi-objective structural optimization for rapid, reliable EM modeling and RF interconnect co-design.

## 2. Key Parameter Analysis and Simulation of Quasi-Coaxial TSV Electromagnetic Performance

In three-dimensional (3D) integrated microsystems, the electromagnetic characteristics of quasi-coaxial through-silicon-via (TSV) composite structures are directly related to system signal integrity and overall performance [15]. This paper investigates the specific effects of key geometric parameters in the composite structure—TSV radius, insulation thickness, and the pitch between signal TSV and ground TSV—on overall transmission performance.

**Figure 1:**
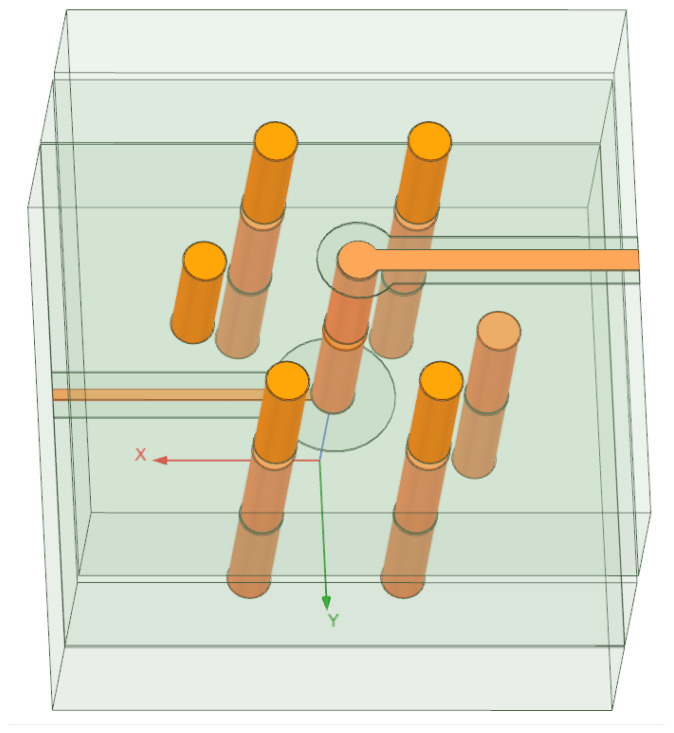
Schematic of the quasi-coaxial composite structure.

The quasi-coaxial TSV composite structure is designed based on silicon-substrate-manufacturing processes and consists of three parts: the upper grounded coplanar waveguide (GCPW) for horizontal signal transmission, the middle quasi-coaxial TSV for vertical interlayer signal interconnection, and the lower silicon-integrated coaxial line (SICL) for further signal distribution and redistribution [16]. As shown in Figure 1, the parameterized model established in ANSYS HFSS clearly illustrates the signal transmission path: the signal departs from the upper GCPW, passes vertically through the quasi-coaxial TSV, and reaches the lower SICL.

**Figure 2:**
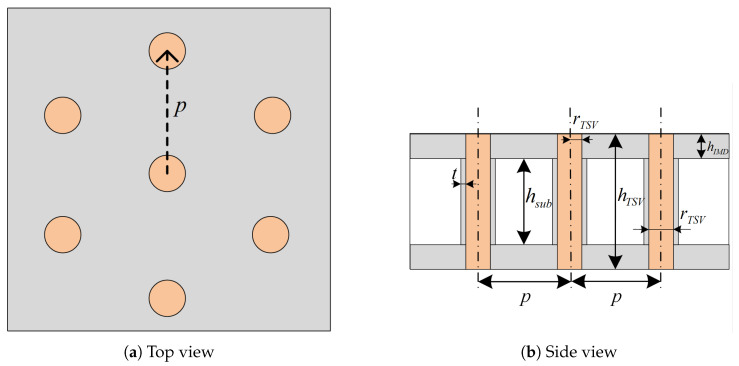
Quasi-coaxial TSV structure schematic.

Figure 2 shows the top and side views of the quasi-coaxial TSV, where the central TSV is the single signal TSV, and six surrounding ground TSVs are evenly distributed. The variables indicated in the figure are defined as follows: *p* denotes the pitch between the signal TSV and the ground TSV, *r* is the TSV radius, $h_{\mathrm{TSV}}$ is the TSV height, *t* is the TSV insulation thickness, $h_{\mathrm{sub}}$ is the substrate height, and $h_{\mathrm{IMD}}$ is the height of the inter-metal dielectric (IMD) layer. In the full-wave simulations, the computational domain was normalized with respect to the in-medium wavelength at the anchor frequency, ensuring that the air-box clearance, PML thickness, port de-embedding distance, and lateral/vertical padding were all set as fractions of the wavelength to suppress boundary artifacts across the entire band. A robustness check confirmed that further enlarging the computational domain produced negligible changes in the *S*-parameter responses, demonstrating that the chosen settings are sufficient to avoid boundary-induced errors.

Simulation results reveal the influence of the key geometric parameters on transmission performance as follows:

(1) Effect of TSV radius

**Figure 3:**
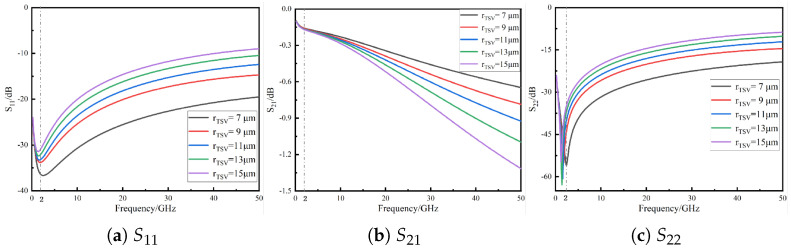
*S*-parameter variation with TSV radius for the quasi-coaxial TSV composite structure.

The TSV radius is one of the key geometric parameters affecting the transmission performance of quasi-coaxial TSV composite structures, as it significantly impacts DC resistance, skin effect, parasitic inductance, and parasitic capacitance [17]. Figure 3 shows the variation in transmission characteristics when the TSV radius changes from 7 μm to 15 μm, with the corresponding $S_{11}$, $S_{21}$, and $S_{22}$ curves plotted in Figure 3a–c. When the operating frequency is below 2 GHz, changes in TSV radius show negligible differences in $S_{11}$ and $S_{22}$, indicating minimal impact on impedance matching. However, above 2 GHz, the skin effect becomes prominent, AC resistance dominates the equivalent resistance of the TSV, and both return loss and insertion loss increase significantly. For instance, at 50 GHz, when the TSV radius increases from 7 μm to 15 μm, $S_{11}$ degrades from −20 dB to −10 dB, while $S_{21}$ increases from −0.6 dB to −1.3 dB.

(2) Effect of TSV insulation thickness

**Figure 4:**
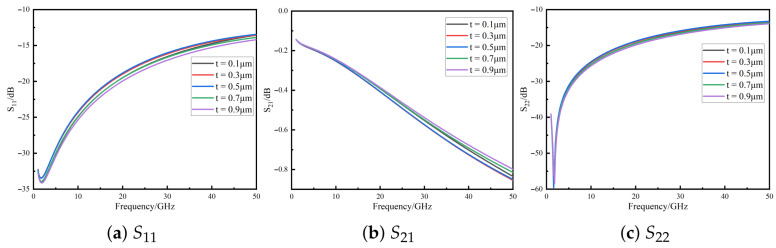
*S*-parameter variation with TSV insulation thickness for the quasi-coaxial TSV composite structure.

The TSV insulation thickness directly influences parasitic capacitance and substrate parasitic conductance [18]. Figure 4 shows the $S_{11}$, $S_{21}$, and $S_{22}$ curves when the insulation thickness varies from 0.1 μm to 0.9 μm. The signal transmission performance exhibits a non-monotonic trend with local fluctuations, indicating the existence of local optima. In the low-frequency range (<1 GHz), the effect is negligible, whereas in the high-frequency range (1–50 GHz), reflection loss and insertion loss first degrade and then improve. At 50 GHz, all insulation thicknesses maintain $S_{11}$ better than −13 dB, highlighting the importance of optimizing insulation thickness for performance enhancement.

(3) Effect of signal-to-ground TSV pitch

**Figure 5:**
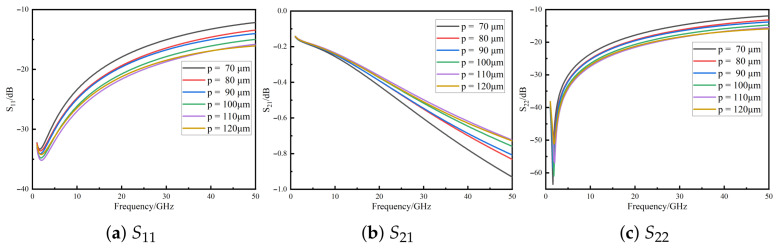
*S*-parameter variation with signal-to-ground TSV pitch for the quasi-coaxial TSV composite structure.

The pitch between the signal and ground TSVs significantly affects parasitic inductance, parasitic capacitance, and substrate parasitic conductance [19]. Figure 5 presents the $S_{11}$, $S_{21}$, and $S_{22}$ curves for pitches ranging from 70 μm to 110 μm. In the high-frequency range (>3 GHz), both $S_{11}$ and $S_{22}$ gradually improve with an increased pitch, but the performance slightly degrades when the pitch exceeds 110 μm. Meanwhile, $S_{21}$ shows significant improvement over the 10–50 GHz range, suggesting that optimal pitch selection is essential for balancing parasitic parameters and preventing performance degradation due to excessive parasitic inductance.

In summary, to enhance the high-frequency signal integrity and transmission performance of the quasi-coaxial TSV composite structure, the TSV radius, insulation thickness, and signal-to-ground pitch should be jointly optimized to control parasitic parameters and achieve optimal performance. These three geometric parameters are, therefore, selected as input variables for constructing the neural network surrogate model.

Based on the determination of design variables, it is necessary to specify the variation ranges of each parameter and adopt an appropriate sampling method to obtain the sample data required for training the neural network. The single-variable analysis presented in the previous section has already defined the variation ranges and step sizes of each variable, as summarized in Table 1. Specifically, the selection of the TSV radius is based on the typical process window and performance optimization requirements [18], while the settings of parameters, such as the insulation layer thickness and the signal-to-ground TSV pitch, are determined with reference to the experimental design results of thermal stress and coupling performance analyses [20].

The three parameters have nine levels each, giving a total design space of 9×9×9=729 possible combinations. Simulating all combinations to obtain training data is impractical. Therefore, an efficient experimental design method is needed to cover the parameter space with minimal samples, thus reducing computational cost while producing high-quality neural network models.

Latin hypercube sampling (LHS) is an efficient experimental design method that performs stratified sampling within a multi-dimensional parameter space and exhibits favorable projection properties. The LHS method ensures that the sample points are uniformly distributed in the projection onto any single dimension, with only one sample point in each projection subinterval. Owing to this property, LHS has been widely applied in uncertainty analysis, deep learning model construction, and other related fields [21].

In this study, the LHS method is employed to generate the training dataset for the neural network model. Given that three input variables are selected and each variable contains nine levels, a total of 81 samples are required to satisfy the uniform distribution requirement in the projection of any two-variable combination. By applying LHS, 81 parameter combinations can be efficiently obtained, with each combination ensuring complete coverage of the sample space in the projection along each individual variable direction.

To avoid data duplication between the training and testing datasets, 81 training samples are first generated using LHS, and then an additional 64 samples are randomly selected from the remaining parameter space (after removing the training set samples) to form an independent testing dataset.

After constructing the training and test sets, 3D full-wave EM simulations are conducted in ANSYS HFSS for all sample points, obtaining broadband two-port *S*-parameter sequences from 1 to 50 GHz with a 200 MHz step. The quasi-coaxial TSV composite structure satisfies reciprocity ($S_{21}=S_{12}$), with port 1 connected to the upper GCPW and port 2 connected to the lower SICL. Thus, only $S_{11}$, $S_{21}$, and $S_{22}$ are used for neural network training and testing.

To further enhance the physical interpretability of the neural network, this study introduces the vector fitting (VF) method to process the broadband S-parameter data obtained from the simulations. Vector fitting is a robust and efficient nonlinear fitting technique that can accurately convert discrete frequency-domain sequence data into a pole–residue form transfer function by appropriately selecting the fitting order [22]. Recent studies have demonstrated that integrating the VF method into parametric neural network modeling can effectively improve the accuracy and generalization capability of complex structure modeling [14]. Its mathematical expression is given as follows:

(1) $$H\left(s\right)=\sum_{i=1}^{N}\frac{r_{i}}{s-p_{i}}$$

where $p_{i}$ and $r_{i}$ can be real or complex–conjugate pairs. This transfer function form is embedded as prior knowledge to guide neural network modeling.

The $S_{11}$, $S_{21}$, and $S_{22}$ data are fitted, achieving errors below −40 dB, demonstrating high accuracy. The fitted orders are 6 for both $S_{11}$ and $S_{21}$, and 5 for $S_{22}$. The pole–residue results include both real and complex–conjugate forms, as summarized in Table 2.

## 3. Causality and Passivity-Constrained Neuro-TF with Particle Swarm Optimization Framework

### 3.1. Neural Network Architecture

**Figure 6:**
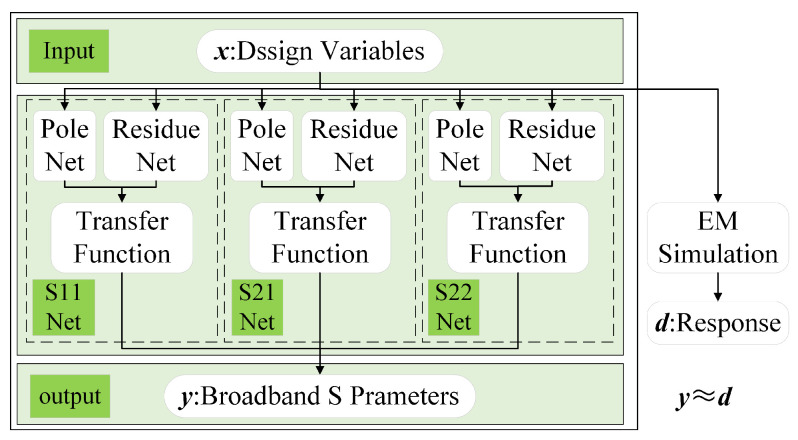
Neural network architecture.

The proposed neural network architecture is shown in Figure 6. Considering that the modeled two-port passive channel is reciprocal ($S_{12}=S_{21}$), the hidden layer contains three parallel sub-networks: the $S_{11}$ network, the $S_{21}$ network, and the $S_{22}$ network. Each sub-network consists of two parts: a pole sub-ANN for predicting poles and a residue sub-ANN for predicting residues. The three sub-networks jointly generate their respective poles and residues, reconstruct the transfer function, and ultimately produce the broadband *S*-parameters at the output layer. The proposed network covers the frequency range of 1–50 GHz with a step size of 200 MHz. Each sub-network adopts two hidden layers with up to 10 neurons per layer to ensure adequate expressive capacity while controlling network complexity.

### 3.2. Training Strategy

**Figure 7:**
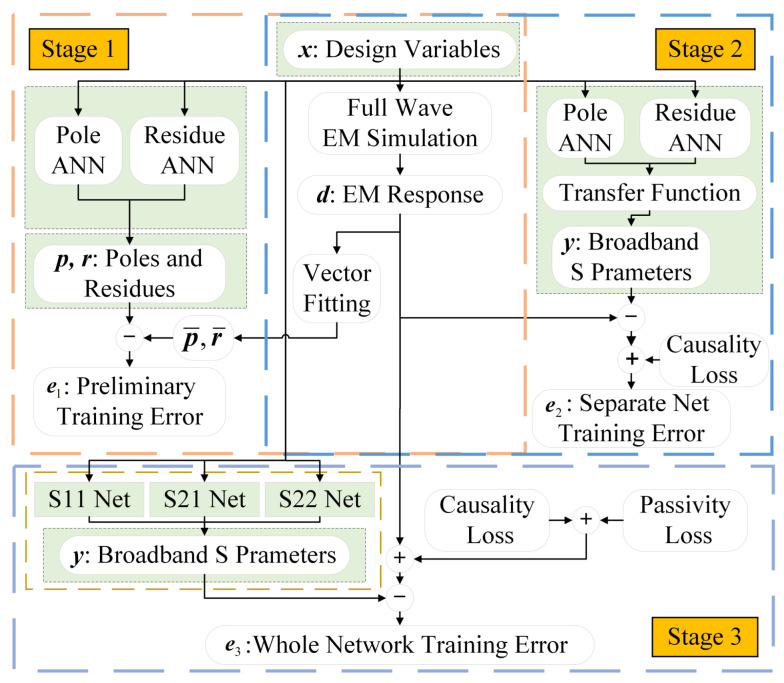
Three-stage training process of the proposed network.

Prior to neural network training, training data are generated through full-wave electromagnetic (EM) simulations, followed by vector fitting (VF) preprocessing. As shown in Figure 7, the entire training process is divided into three stages:

Prior to neural network training, the training data are generated through full-wave electromagnetic (EM) simulations, followed by vector fitting (VF) preprocessing. As illustrated in Figure 7, the entire training process is divided into three stages.

- **Stage 1:** In the first stage, the pole sub-network and the residue sub-network are trained separately to learn the mapping relationships between the design variables and the pole–residue parameters, using the mean squared error (MSE) as the loss function for optimization. For complex–conjugate pairs of poles and residues, only one element of each pair is trained, while the other is constructed through the conjugate relationship. This strategy simplifies the network architecture design and strictly preserves the mathematical complex–conjugate symmetry. Since multiple pole and residue sub-networks need to be trained, manually tuning the hyperparameters of each network—such as the learning rate, the number of hidden layers, and the number of neurons per layer—requires extensive trial-and-error to identify the optimal hyperparameter combination [23]. To accelerate this process, Bayesian optimization is integrated with the neural network training, taking the learning rate, the number of hidden layers, and the number of neurons per layer as the optimization parameters. Their search ranges are predefined, and the maximum number of iterations is set. By constructing a black-box model, the best-performing hyperparameter combination and the corresponding network are identified through multiple iterations [24]. The selected hyperparameters and their corresponding values from the Bayesian optimization process are summarized in Table 3. In addition to causality and passivity, reciprocity can also be consistently enforced in the surrogate when the physical stack is reciprocal, either as a hard structural constraint ($R_{k}=R_{k}^{T}$, $D=D^{T}$, $S_{12}\equiv S_{21}$) or as a soft regularizer $L_{\mathrm{rec}}$, thereby ensuring symmetric responses across ports.
- **Stage 2:** The main $S_{11}$, $S_{21}$, and $S_{22}$ networks are trained individually, loading the pretrained weights from Stage 1. The aim is to reduce *S*-parameter prediction errors while enforcing causality, i.e., all poles must lie in the left-half complex plane.
- **Stage 3:** End-to-end training is performed, introducing passivity constraints as additional objectives. In all stages, the prediction accuracy loss term is defined as $L_{\mathrm{fit}}$ (MSE).

Two key physical constraints—causality and passivity—are incorporated as regularization terms in the loss function to ensure physically consistent frequency-domain responses. Their design and implementation are described as follows:

#### 3.2.1. Causality Constraint Loss

Causality requires that all poles reside in the left-half complex plane. The constraint is formulated as follows:

(2) $$L_{\mathrm{cau}}=\frac{1}{N}\sum_{i=1}^{N}\mathrm{ReLU}\left(ℜ(p_{i})\right),$$

where $p_{i}$ is the *i*-th pole, and ℜ(·) denotes the real part. If a pole lies in the right-half plane ($ℜ(p_{i})>0$), the ReLU term imposes a penalty to drive it back into the left-half plane. The Stage 2 total loss is as follows:

(3) $$L_{\mathrm{separate}}=L_{\mathrm{fit}}+L_{\mathrm{causal}},$$

#### 3.2.2. Passivity Constraint

In the frequency domain, passivity is equivalent to the *S*-parameter matrix, satisfying the inequality (for any f∈Ω) [7]:

(4) $$S^{*}\left(f\right)S\left(f\right)\leq I,$$

where $S^{*}\left(f\right)$ is the conjugate transpose of S(f). Performing singular value decomposition (SVD) on S(f) yields singular values $\sigma_{1}\left(f\right),\sigma_{2}\left(f\right),\ldots$. Passivity requires the following:

(5) $$\sigma_{1}\left(f\right)\leq 1,\;\forall f\in \Omega$$

The passivity penalty term is defined as follows:

(6) $$L_{\mathrm{passive}}=\frac{1}{N}\sum_{i=1}^{N}\mathrm{ReLU}\left(\sigma_{1}\left(f_{i}\right)-1\right)$$

imposing a penalty only when the maximum singular value exceeds 1.

#### 3.2.3. Stage 3 Total Loss

The Stage 3 objective function integrates three terms:

(7) $$L_{\mathrm{whole}}=L_{\mathrm{fit}}+L_{\mathrm{causal}}+L_{\mathrm{passive}}$$

This ensures that the network output is both accurate and physically consistent. After Stage 3, the trained network accurately models the frequency-domain behavior of multi-port interconnects while satisfying causality and passivity.

### 3.3. Electrical Performance Optimization of Particle Swarm Optimization Under Loss Constraints

In practical systems, various performance parameters are often coupled and mutually influential [25]. Traditional manual optimization methods typically fix certain parameters while adjusting only a single parameter, resulting in solutions that are merely suboptimal under specific conditions. Such solutions may not represent the global optimum and may even fail to be locally optimal [26]. When the input parameter dimensionality is high and the search space is large, relying on human expertise for multi-objective, multi-parameter joint optimization poses significant challenges and makes it difficult to effectively obtain the optimal parameter combination [27].

The introduction of intelligent optimization algorithms, such as genetic algorithms and particle swarm optimization, provides an efficient and feasible approach to address such problems [28,29]. By defining optimization objectives, specifying design variables, and establishing optimization criteria, intelligent optimization methods can automatically search for the optimal solution in high-dimensional design spaces, thereby significantly improving the efficiency and quality of multi-objective optimization [30].

As a population-based stochastic optimization technique, PSO requires reasonable allocation of weights among multiple objectives to reflect their relative importance when handling multi-objective problems. Higher weights can be assigned to critical objectives, while lower weights are given to secondary ones. This approach balances potential conflicts among objectives during optimization and enhances both the convergence and stability of the search process. The setting of optimization weights forms the core of the optimization criteria and is key to achieving multi-objective coordination and efficient problem solving.

In this study, the optimization variables are the frequency-domain performance parameters $S_{11}$, $S_{21}$, and $S_{22}$ of the quasi-coaxial TSV composite structure. To prevent different objective ranges from biasing the search, we normalize the deviations from prescribed targets and define the scalarized fitness as follows:

Let the (dB) deviations from the target values be

(8) $$\Delta S_{11}:=\left|S_{11}-S_{11,\mathrm{target}}\right|,\;\Delta S_{21}:=\left|S_{21}-S_{21,\mathrm{target}}\right|,\;\Delta S_{22}:=\left|S_{22}-S_{22,\mathrm{target}}\right|.$$

For each objective ij∈{11,21,22}, we apply a min–max normalization over the current population:

(9) $${\tilde{\Delta S}}_{ij}\;:=\;\frac{\Delta S_{ij}-{\left(\Delta S_{ij}\right)}_{\min}}{{\left(\Delta S_{ij}\right)}_{\max}-{\left(\Delta S_{ij}\right)}_{\min}+\epsilon },\;\epsilon >0\;(e.g.,\;10^{-12}),$$

where ${\left(\Delta S_{ij}\right)}_{\min}$ and ${\left(\Delta S_{ij}\right)}_{\max}$ denote the minimum and maximum deviations within the current generation.

Using the same weight symbols as in the text, the normalized fitness is

(10) $$f\;\equiv \;L\;=\;\alpha \;{\tilde{\Delta S}}_{21}+\beta \;{\tilde{\Delta S}}_{11}+\gamma \;{\tilde{\Delta S}}_{22},\;\alpha +\beta +\gamma =1.$$

Here, *f* is the normalized fitness; α, β, and γ are the weights for insertion loss $S_{21}$ and return losses $S_{11}$/$S_{22}$, respectively. This unifies the formula with the convex scalarization $L=\alpha \;\ell \left(S_{21}\right)+\beta \;\ell \left(S_{11}\right)+\gamma \;\ell \left(S_{22}\right)$ by setting $\ell \left(S_{ij}\right)={\tilde{\Delta S}}_{ij}$.

To improve end-to-end link performance under broadband objectives, we set α=0.5 and β=γ=0.25. This reflects that $|S_{21}|$ (in dB) enters the link budget and dynamic range linearly, thus exerting a first-order impact on SNR/BER; by contrast, $|S_{11}|$ and $|S_{22}|$ mainly act through reflections/standing waves, for which incremental benefits diminish once return loss is better than the common −10 dB threshold. Under normalized objectives, the higher weight on $S_{21}$ suppresses broadband insertion-loss magnitude and ripple, while the combined 0.5 weight on $S_{11}$/$S_{22}$ prevents single-metric bias. We adopt a 40 GHz anchor to set the targets for reporting and guidance:

$$S_{11,\mathrm{target}}=-25\;\mathrm{dB},\;S_{21,\mathrm{target}}=-0.54\;\mathrm{dB},\;S_{22,\mathrm{target}}=-24\;\mathrm{dB}.$$

Small adjustments of α around 0.5 move the solution along the Pareto front (larger α favors lower insertion loss/ripple; smaller α strengthens matching).

All surrogate poles are restricted to the left-half plane (causality), and the maximum singular value satisfies $\sigma_{\max}\left(f\right)\leq 1$ over 1–50 GHz (passivity); reciprocity can be optionally enforced for reciprocal stacks.

To balance global exploration and local exploitation, we adopt a linearly decreasing inertia weight:

(11) $$\omega \left(t+1\right)=\left(\frac{\max\text{\_}\mathrm{iter}-t}{\max\text{\_}\mathrm{iter}}\right)\left(\omega_{\max}-\omega_{\min}\right)+\omega_{\min},$$

where max_iter is the maximum iteration count, *t* is the current iteration index, and $\omega_{\max}$/$\omega_{\min}$ are the maximum/minimum inertia weights. A larger initial ω enhances global search in early stages; as *t* increases, ω decreases to strengthen local refinement.

The parameters of the particle swarm optimization (PSO) algorithm and their values or ranges are shown in Table 4.

After clearly defining the design parameters involved in the PSO algorithm and their value ranges, the multi-objective and multi-parameter intelligent optimization process can be carried out. In the initialization stage, the three design variables—TSV radius, TSV insulation thickness, and the pitch between the signal TSV and the ground TSV—are first assigned initial values, and the optimization objective function is set. Subsequently, the PSO algorithm initializes the initial positions and velocities of all particles in the population, where the position vector $X=[X_{1},X_{2},X_{3}]$ corresponds to the values of the three design parameters, and the velocity vector $V=[V_{1},V_{2},V_{3}]$ represents their adjustment step sizes in the design space.

**Figure 8:**
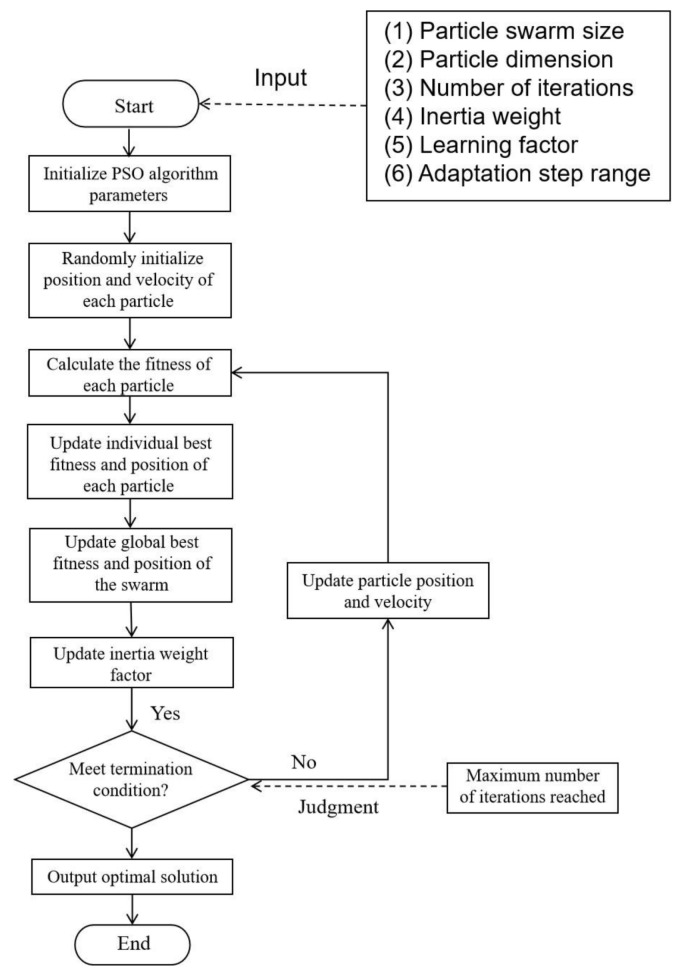
PSO optimization process.

The proposed multi-objective optimization framework integrates the PSO algorithm with the proposed neural network model, both implemented in PyTorch 2.8.0. The specific PSO optimization process is shown in Figure 8.

During subsequent iterations, the PSO algorithm continuously updates the three selected optimization variables and ensures they always remain within the preset value ranges. In each iteration, the updated parameters are input into the neural network surrogate model of the quasi-coaxial TSV composite structure to rapidly predict the *S*-parameter sequence within the corresponding frequency band. The PSO algorithm then extracts the *S*-parameter values at specific frequency points and calculates the fitness value of each particle.

By comparing the fitness values of all particles in the current iteration, the local optimal solution is determined—that is, the particle with the minimum fitness value in this iteration, along with its corresponding parameter combination and objective function value. This local optimal solution is further compared with the historically recorded global optimal solution: if the current local optimal solution has a better fitness value than the global optimal solution, the global optimal solution is updated; otherwise, the original global optimal solution remains unchanged.

When the number of iterations reaches the preset maximum, the optimization process terminates. The design parameters corresponding to the final global optimal solution, namely the TSV radius, TSV insulation thickness, and the pitch between the signal TSV and the ground TSV, are taken as the optimal geometric configuration of the structure under multi-objective constraints.

## 4. Results and Analysis

To evaluate the effectiveness of the proposed framework, we first validated the neural surrogate on a training set of 81 samples (LHS) and a test set of 64 random samples. The design variables are summarized in Table 5. The network achieved average prediction errors of 1.93% ($S_{11}$), 2.29% ($S_{21}$), and 2.11% ($S_{22}$), showing good agreement with FEM results. After training, both causality and passivity errors converged to zero, confirming that the surrogate strictly respects the physical constraints. To further validate generalization under extreme conditions, 15 corner/face-center/center cases (8 corners, 6 face-centers, 1 center) were simulated and compared against surrogate predictions. The broadband RMS/Max/95th-percentile errors of $|S_{21}|$ and $|S_{11}|$ remained within engineering tolerance, and passivity was preserved throughout the 1–50 GHz band.

**Figure 9:**
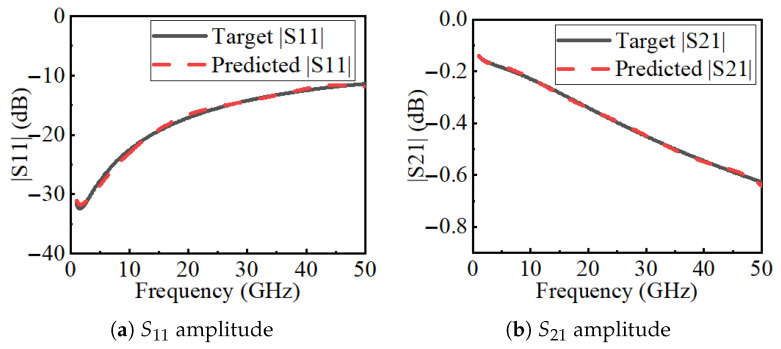
Comparison of S-parameters predicted by FEM and the neural surrogate.

Figure 9 compares the *S*-parameters predicted by FEM and by the neural surrogate. The two sets of curves agree well, showing that the surrogate accurately reproduces both reflection and transmission responses across the band.

**Figure 10:**
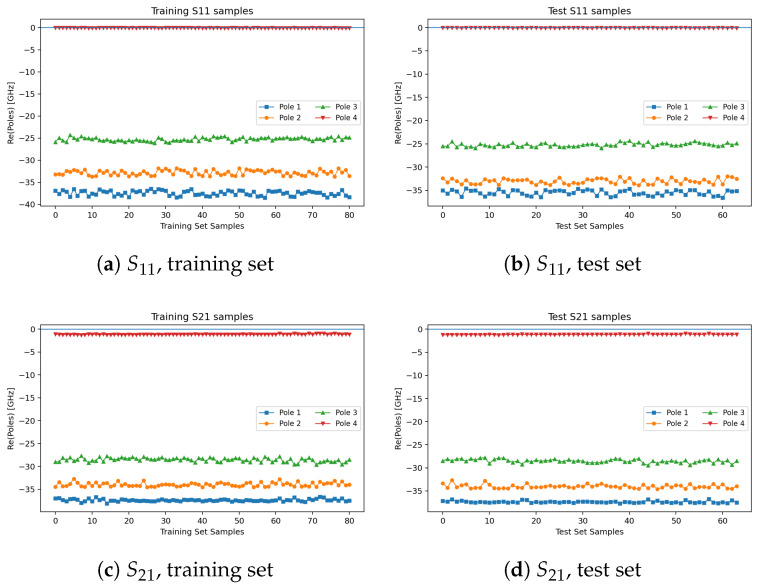
Real-part distributions of the poles of $S_{11}$ and $S_{21}$ (training vs. test).

**Figure 11:**
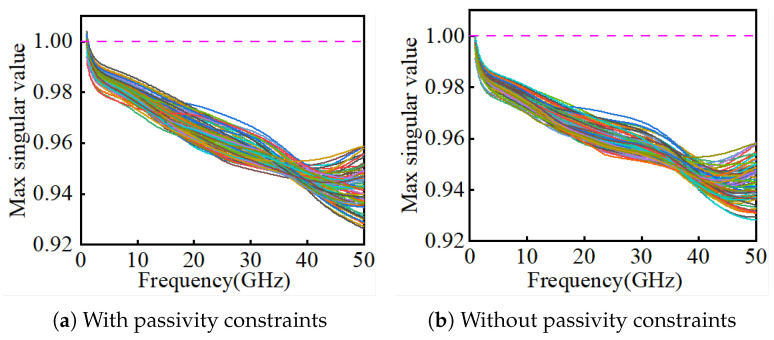
Comparison of largest singular values of NN-predicted S-parameters.

Figure 10 illustrates the distributions of the real parts of the poles of $S_{11}$ and $S_{21}$, confirming that all poles lie in the left-half plane (causality satisfied). Figure 11 compares singular values with and without the passivity constraint: without constraints, violations $\sigma_{\max}>1$ occur; with constraints, $\sigma_{\max}\leq 1$ is always maintained.

**Figure 12:**
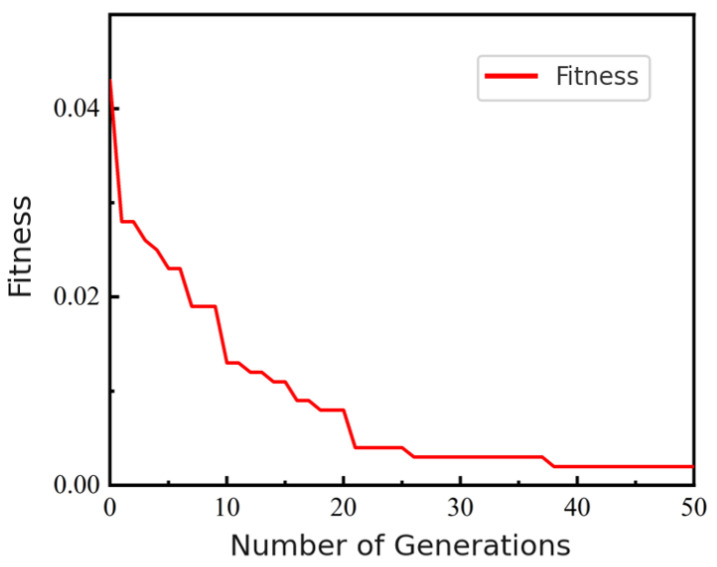
Evolution of minimum population fitness in PSO.

**Figure 13:**
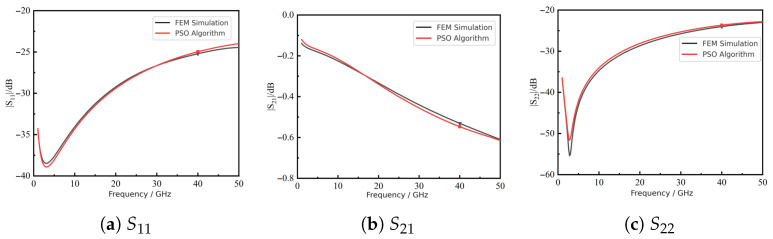
Comparison of *S*-parameters between PSO-rounded predictions and FEM results.

Next, we integrated the surrogate with PSO for multi-objective optimization. Figure 12 shows that the fitness converged after about 26 iterations, reaching a minimum of 0.0021 at the 50th iteration. The best solution was r=7μm, t=0.86μm, p=110μm; for fabrication convenience, *t* was rounded to 0.90 μm. Figure 13 compares the predicted S-parameters of this rounded design with FEM results, showing close agreement.

At 40 GHz, Table 6 shows that deviations from the optimization targets were within 3% for all *S*-parameters, confirming the accuracy of the framework. Table 7 further compares PSO predictions with FEM simulations, with absolute errors below 0.84 dB. The 40 GHz marker in Figure 13 illustrates the anchor for solution selection; broadband consistency is ensured by training and auditing over the entire 1–50 GHz band.

Finally, Table 8 highlights the efficiency of the proposed approach. Under an equal budget of 50 FEM calls, the FEM-only optimization required 8085 s and 8.6 GB, whereas the surrogate–PSO framework required only 637 s and 2.5 GB, saving over 90% in runtime and 70% in memory. For fairness, the budget of 50 high-fidelity FEM calls was chosen as the median of the typical 40–60 runs required in conventional workflows (initial scans plus iterative refinements). Internal iterations of the surrogate/PSO are not counted toward the expensive cost, and both schemes are evaluated under the same FEM budget.

## 5. Conclusions

The main research work and innovative contributions of this paper are as follows:

1. **Structure modeling and parameter mechanisms.** For the silicon–substrate *quasi-coaxial TSV–GCPW–SICL* interconnect, full-wave HFSS simulations over 1–50GHz confirm clear design trends. Increasing the TSV radius from 7 to 15μm degrades matching and insertion: at 50GHz, $S_{11}$ changes from about −20dB to −10dB and $S_{21}$ from about −0.6dB to −1.3dB. Varying the insulation thickness within the studied range maintains $S_{11}<-13\;\mathrm{dB}$ at 50GHz. Enlarging the signal–ground TSV pitch (around 70–110μm) improves transmission in 10–50GHz, while exceeding ∼110 μm slightly worsens matching $\left(S_{11}/S_{22}\right)$. These observations support joint tuning of the radius, insulation thickness, and pitch for broadband, low-loss interconnects.
2. **Physics-constrained Neuro-TF surrogate.** The proposed model achieves average test errors of 1.93% $\left(S_{11}\right)$, 2.29% $\left(S_{21}\right)$, and 2.11% $\left(S_{22}\right)$. After training, all real pole parts lie in the left half-plane, and the largest singular value satisfies $\sigma_{\max}\;\left(S(f)\right)\leq 1$ across the band, indicating compliance with *causality* and *passivity*. Together with the three-stage training strategy, this yields physically consistent, broadband prediction from 1–50GHz.
3. **PSO-based multi-objective co-optimization.** With 40GHz targets, $S_{11}=-25\;\mathrm{dB}$, $S_{21}=-0.54\;\mathrm{dB}$, and $S_{22}=-24\;\mathrm{dB}$ (weights α=0.5,β=γ=0.25), and a linearly decreasing inertia weight, the minimum fitness reaches 0.0021 at the 50th generation. The corresponding (rounded) geometry includes r=7μm,t=0.9μm, and p=110μm. At 40GHz, deviations from the targets are $S_{11}=-24.37\;\mathrm{dB}$ (0.63dB,2.5%), $S_{21}=-0.558\;\mathrm{dB}$ (0.018dB,3.3%), and $S_{22}=-23.28\;\mathrm{dB}$ (0.72dB,3.0%). Relative to FEM at the same frequency, the discrepancies are 0.84dB $\left(S_{11},\;3.3\%\right)$, 0.019dB $\left(S_{21},\;3.5\%\right)$, and 0.74dB $\left(S_{22},\;3.1\%\right)$. Under the same iteration budget, the NN–PSO framework reduces the runtime from 8085s to 637s (−92.1%) and memory from 8.6GB to 2.5GB (−70.9%) compared with direct FEM-only optimization.
4. **Novelty and impact.** Compared with previous works (e.g., purely data-driven surrogates with post hoc passivization, single-frequency or single-metric optimization, or commercial black-box optimizers), this study introduces a physics-constrained pole–residue neural surrogate and an external multi-objective PSO loop that jointly enforce causality, passivity (and optional reciprocity), and multi-port consistency. This combination shrinks the hypothesis space, stabilizes convergence with only ∼50 FEM calls, and outputs an auditable Pareto front that balances broadband insertion loss, return losses, and ripple under fabrication constraints. The framework thus provides not only accurate and efficient optimization for quasi-coaxial TSVs but also a transferable methodology for other broadband passive interconnects.

## Figures and Tables

**Table 1:** Design variable information for the quasi-coaxial TSV composite structure case.

Variable	Symbol	Min	Max	Step	Unit
TSV radius	*r*	7	15	1	μm
TSV insulation thickness	*t*	0.1	0.9	0.1	μm
Signal-to-ground TSV pitch	*p*	70	110	5	μm

**Table 2:** Pole and residue characteristics of *S*-parameters obtained from vector fitting.

Parameter	Order	Real Poles	Complex-Conjugate Pairs
$S_{11}$	6	2	2
$S_{21}$	6	2	2
$S_{22}$	5	1	2

**Table 3:** Hyperparameters selected by Bayesian optimization.

Hyperparameter	Min	Max	Type
Learning rate	$1\times 10^{-5}$	0.05	Continuous
Hidden layers	1	2	Integer
Neurons per layer	3	8	Integer

**Table 4:** Details of the PSO parameters.

Parameter	Symbol	Value or Range
Maximum iterations	*max_iter*	50
Inertia weight factor	ω	[0.1, 1]
Acceleration coefficient	$C_{1}$	2
	$C_{2}$	2
Population size	*N*	50
Particle position	$X_{1}$	[7, 15]
	$X_{2}$	[0.1, 0.9]
	$X_{3}$	[70, 110]
Particle velocity	$V_{1}$	[−1, 1]
	$V_{2}$	[−0.01, 0.01]
	$V_{3}$	[−1, 1]

**Table 5:** Design parameters of the training set.

Design Variable	Symbol	Min	Max	Step	Unit
TSV radius	$r_{\mathrm{TSV}}$	7	15	1	μm
Insulation thickness	$t_{\mathrm{ox}}$	0.1	0.9	0.1	μm
TSV pitch	*p*	70	110	5	μm

**Table 6:** Comparison between PSO optimization targets and predicted values at 40 GHz.

Parameter	Target	Predicted	Rel. Error	Abs. Error
$S_{11}$	−25 dB	−24.37 dB	2.5%	0.63 dB
$S_{21}$	−0.54 dB	−0.558 dB	3.3%	0.018 dB
$S_{22}$	−24 dB	−23.28 dB	3.0%	0.72 dB

**Table 7:** Comparison between PSO predictions and FEM results at 40 GHz.

Parameter	PSO Prediction	FEM Result	Rel. Error	Abs. Error
$S_{11}$	−24.37 dB	−25.21 dB	3.3%	0.84 dB
$S_{21}$	−0.558 dB	−0.539 dB	3.5%	0.019 dB
$S_{22}$	−23.28 dB	−24.02 dB	3.1%	0.74 dB

**Table 8:** Comparison of computational resources between FEM-only and surrogate–PSO optimization.

Resource	FEM (50 Sims)	Surrogate–PSO	Improvement
Time	8085 s	637 s	92.1%
Memory	8.6 GB	2.5 GB	70.9%

## Data Availability

Data are contained within the article.

## Abbreviations

The following abbreviations are used in this manuscript:

TSV	Through-Silicon-Via
GCPW	Grounded Coplanar Waveguide
SICL	Silicon-Integrated Coaxial Line
EM	Electromagnetic
RF	Radio Frequency
VF	Vector Fitting
PSO	Particle Swarm Optimization
ANN	Artificial Neural Network
SVD	Singular Value Decomposition
MSE	Mean Squared Error
LHS	Latin Hypercube Sampling
IMD	Inter-Metal Dielectric
FEM	Finite Element Method
